# RANK/RANKL/OPG Signalization Implication in Periodontitis: New Evidence from a RANK Transgenic Mouse Model

**DOI:** 10.3389/fphys.2017.00338

**Published:** 2017-05-24

**Authors:** Bouchra Sojod, Danielle Chateau, Christopher G. Mueller, Sylvie Babajko, Ariane Berdal, Frédéric Lézot, Beatriz Castaneda

**Affiliations:** ^1^INSERM, UMR-1138, Laboratoire de Physiopathologie Orale Moléculaire, Centre de Recherche des Cordeliers Paris, France; ^2^INSERM, UMR-1138, Intestine: Nutrition, Barrier, and Diseases Group, Centre de Recherche des Cordeliers Paris, France; ^3^Laboratoire Immunologie et Chimie Thérapeutiques, Centre National de la Recherche Scientifique, UPR-9021, Institut de Biologie Moléculaire et Cellulaire, Université de Strasbourg Strasbourg, France; ^4^INSERM, UMR-957, Laboratoire de Physiopathologie de la Résorption Osseuse et Thérapie des Tumeurs Osseuses Primitives, Faculté de Médecine, Université de Nantes Nantes, France; ^5^Department of Basic Studies, Faculty of Odontology, University of Antioquia Medellin, Colombia

**Keywords:** periodontitis, RANK, osteoclasts, alveolar bone, root resorption, gingival epithelium, malassez epithelial rests (MER)

## Abstract

Periodontitis is based on a complex inflammatory over-response combined with possible genetic predisposition factors. The RANKL/RANK/OPG signaling pathway is implicated in bone resorption through its key function in osteoclast differentiation and activation, as well as in the inflammatory response. This central element of osteo-immunology has been suggested to be perturbed in several diseases, including periodontitis, as it is a predisposing factor for this disease. The aim of the present study was to validate this hypothesis using a transgenic mouse line, which over-expresses RANK (R^Tg^) and develops a periodontitis-like phenotype at 5 months of age. R^Tg^ mice exhibited severe alveolar bone loss, an increased number of TRAP positive cells, and disorganization of periodontal ligaments. This phenotype was more pronounced in females. We also observed dental root resorption lacunas. Hyperplasia of the gingival epithelium, including Malassez epithelial rests, was visible as early as 25 days, preceding any other symptoms. These results demonstrate that perturbations of the RANKL/RANK/OPG system constitute a core element of periodontitis, and more globally, osteo-immune diseases.

## Introduction

Periodontitis (Pd) is a complex condition with a known multifactorial etiology. A bacterial infection first causes an inflammatory response of periodontal tissues and subsequent periodontal ligament detachment from the cementum, alveolar bone resorption, and gingival recession (Loe et al., 1965; Kinane et al., 2005). A correlation between Pd and systemic bone metabolic diseases (BMD) has been established based on observations realized in clinical trials (Renvert, 2003; Sundaram et al., 2013). Periodontal bone resorption is induced by osteoclasts. Receptor activator of nuclear factor-κB ligand (RANKL), its receptor RANK, and a decoy receptor osteoprotegerin (OPG) are key molecules that regulate osteoclast differentiation, recruitment, and function (Lacey et al., 1998; Suda et al., 1999). This signaling pathway is important for the development and maintenance of periodontal ligament (Hasegawa et al., 2002; Wise et al., 2002, 2003; Mogi et al., 2004; Vernal et al., 2004; Kawasaki et al., 2006; Lu et al., 2006; Nishijima et al., 2006). Under physiological conditions, RANK and RANKL are expressed in dental follicles during tooth eruption and periodontal ligament during adulthood (Sokos et al., 2015). OPG is an important homeostatic control factor of periodontal ligament (Sakata et al., 1999; Wada et al., 2001; Hasegawa et al., 2002; Wise et al., 2002, 2003) and protects the cementum against root resorption (Liu et al., 2016).

An increased RANKL/OPG ratio has been reported in periodontal tissues under pathological conditions, such as Pd (Mogi et al., 2004). RANKL levels in periodontal fibroblasts are induced either by mechanical forces or bacterial challenge in periodontitis (Bostanci et al., 2007), whereas OPG levels decrease under similar conditions (César-Neto et al., 2007). The OPG null mutant mouse, which exhibits alveolar bone loss (Koide et al., 2013) and early onset root resorption (Liu et al., 2016), was recently reported as a model of Pd using the dental ligature procedure (Mizuno et al., 2015). This study validated the importance of the RANKL/RANK/OPG signaling pathway in the physiopathology of Pd.

We have reported the importance of the RANKL/RANK/OPG signaling system in dento-alveolar development in previous studies (Castaneda et al., 2011, 2013; Gama et al., 2015). RANK overexpression causes an increase in the number of TRAP positive cells in alveolar bone and early tooth eruption associated with accelerated root elongation in young mice. However, the effects of RANK overexpression in adult mice and Pd have not yet been studied. The aim of the present study was to analyze the periodontal phenotype of the R^Tg^ mouse to determine the role of the RANKL/RANK/OPG triad as a predisposing factor for Pd.

## Materials and methods

### Animal model

R^Tg^ mice on a CD-1 background, overexpressing RANK under the control of the MRP8 promoter (Castaneda et al., 2011; Duheron et al., 2011), were crossbred, reproduced, and euthanized by people certified for animal experimentation following the protocols validated by the management of the veterinarian services (Ministry of Agriculture of France: agreement #01083.02). The mouse genotypes were determined by PCR using 100 ng of genomic DNA extracted from the tail of each animal and a set of primers for RANK transgene amplification (Fwd: ATG GAC TAC AAA GAC GAT GAC GAC, Rev: TGC CAG GAT CCA CCG CCA CCA). The resulting 320 bp R^Tg^ amplicons indicated the presence of the expected transgene. Each analysis was performed on control and transgenic mice from the same litter for comparison.

### Micro-CT analyses

Analyses of bone microarchitecture were performed using a Skyscan 1076 *in vivo* micro-CT scanner (Skyscan, Kontich, Belgium). Tests were performed after euthanizing the mice for each group. All heads were scanned using the same parameters (pixel size 18 μm, 50 kV, 0.5-mm Al filter, 10 min of scanning). The reconstruction was analyzed using NRecon and CTan software (Skyscan). Three-dimensional visualizations of the heads were performed using ANT software (Skyscan).

### Histology

Heads were collected from euthanized mice and fixed in 4% buffered paraformaldehyde (PFA) in phosphate buffered saline 0.1 M (PBS) for 48 h. Heads were decalcified in 4.13% EDTA/0.2% PFA pH 7.4 in PBS for 4 days in a KOS sw10 (Milestone, Sorisole, Italy). The samples were dehydrated and embedded in paraffin or maintained in a PBS buffer solution at 4°C before cryostat sectioning. Then, 3-μm-thick frontal sections stained with Hematoxylin-eosin and Masson's trichrome (three-color staining protocol, which stains muscle fibers in red, collagen and bone in green, cytoplasm in light red, and nuclei in dark brown) were observed using a DMRXA microscope (Leica, Nussloch, Germany). Tartrate resistant acid phosphatase (TRAP) staining was performed as previously described (Castaneda et al., 2011) to identify the multinucleated osteoclast cells after a 90 min incubation in a 1 mg/mL Naphtol AS-TR phosphate, 60 mmol/L N,Ndimethylformamide, 100 mmol/L sodium tartrate, and 1 mg/mL Fast red TR salt solution (all from Sigma Chemical Co., St Louis, MO, USA) and counterstained with hematoxylin.

### Immunofluorescence

Tissues mounted on Freeze Gel (Labonord, Z.I. de Templemars, France) were used for cryostat sections. The decalcified mouse heads were immersed sequentially in 15 and 30% sucrose in PBS. Sections were air dried and then saturated with 1% BSA in PBS for 30 min to block nonspecific binding sites. Slides were incubated with a rabbit polyclonal primary antibody directed against Keratin 14 (Covance AF64, Princeton, NJ, USA) diluted 1/500 in PBS at room temperature for 1 h. After rinsing three times with PBS, the sections were incubated with a secondary goat polyclonal anti-rabbit IgG antibody coupled to Alexa Fluor 594 (A-11072, Life Technologies) at room temperature for 1 h and then rinsed and incubated for 10 min with DAPI (4,6-Diamidino-2-phenylindole dihidrochloride). After rinsing with PBS, the slides were mounted with cover slips and the fluorescence-mounting medium, Fluoprep (BioMérieux, Marcy l'Etoile, France). DAPI staining was used to evaluate the cell density.

### Transmission electron microscopy (TEM)

The mandibular fragments containing the first molar with surrounding periodontal tissues were fixed at room temperature for 24 h with Karnovsky's solution (at a final concentration of 2% PFA and 2.5% glutaraldehyde in 0.24 M Sörensen's phosphate buffer) and decalcified in 10% EDTA (pH 7.2) for 2 weeks. Specimens were washed in 0.1 M sodium cacodylate (pH 7.2) and transferred to cacodylate-buffered 1% osmium tetroxide at pH 7.2 and incubated for 1.5 h at room temperature. After again washing with 0.1 M sodium cacodylate, samples were treated with 0.5% uranyl acetate for 2 h and dehydrated in graded ethanol, then embedded in Spurr. Semi-thin sections, stained with 1% toluidine blue, were examined under the light microscope. Suitable regions were carefully selected for trimming of the blocks. Eighty-nanometers ultrathin sections were cut and stained with 1% uranyl acetate and lead citrate, and observed by TEM (JEM-1200EX, Japan).

### Statistical analysis

Statistical analyses were performed using GraphPad Prism (GraphPad Software, Inc. San Diego, USA). Descriptive statistics were calculated, and values were given as the means ± standard deviation (SD) of at least five experiments. For statistical validation of the data obtained by measurements, a non-parametric Mann-Whitney test was employed to compare the means of the variables. Significance was defined when *P* < 0.01.

## Results

### Rank overexpression induces severe alveolar bone loss

**Figure 1:**
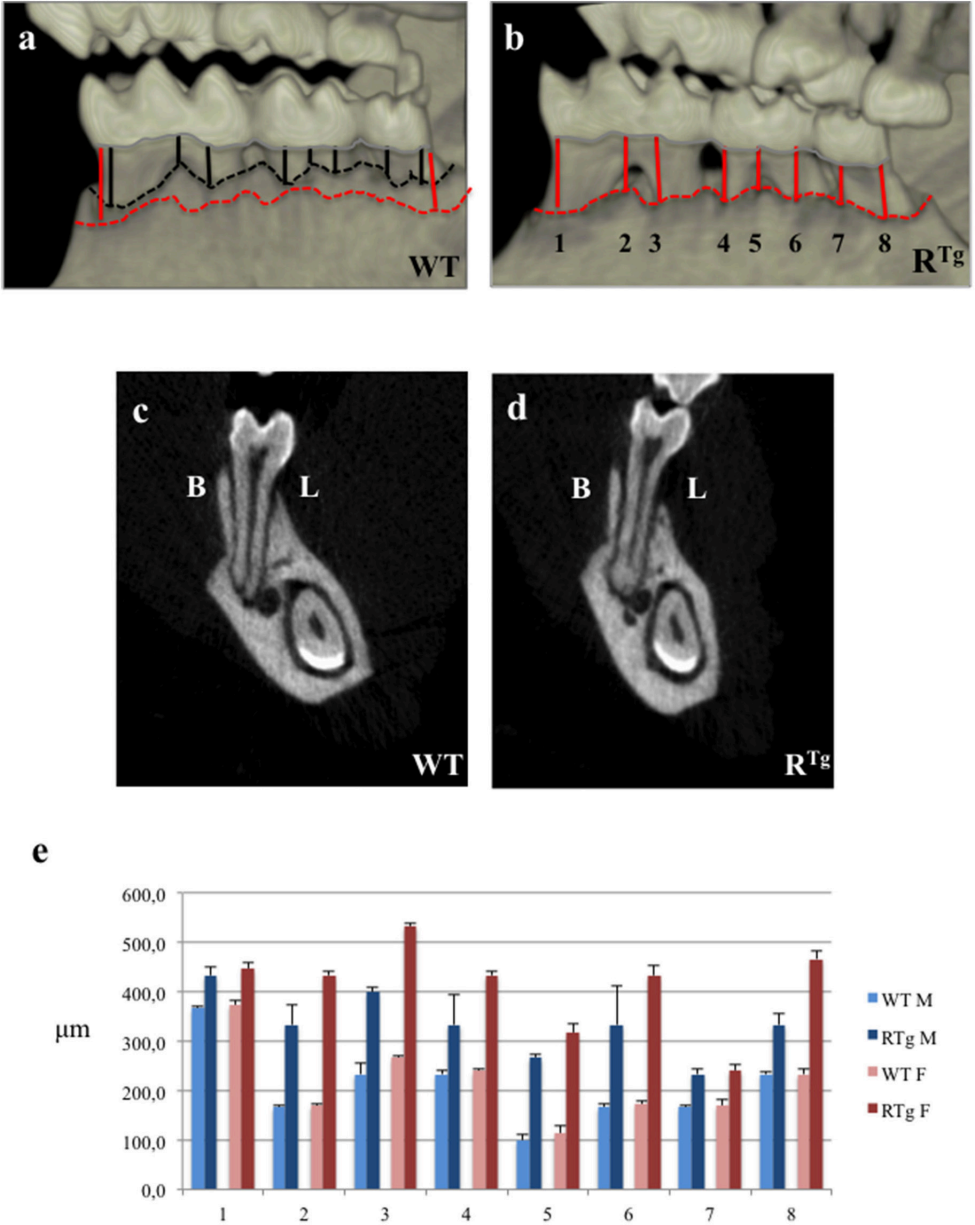
**Micro-Computed-Tomography (micro-CT) analysis of alveolar bone height in 5-month-old mice**. Distances between the cervical enamel and alveolar bone crest of the mandible were measured for each molar in eight positions (black lines for WT mice, and red lines for R^Tg^ mice). We observed a loss in height in R^Tg^ mice relative to WT mice, in the interproximal and inter-radicular areas **(a,b)**, as well as the buccal (B) and lingual (L) alveolar crests in frontal sections **(c,d)**. The mean distances between alveolar bone crest and cervical enamel (μm) were measured in eight different areas **(e)**. Statistical analyses showed significant differences (*p* < 0.01), with greater bone loss in R^Tg^ mice (*n* = 10), which was greater in transgenic females (*n* = 5).

We performed a comparative micro-CT analysis of wild type (WT) and R^Tg^ mice to study the consequence of RANK overexpression on alveolar bone (Figure 1). We observed a substantial reduction of alveolar bone height in R^Tg^ mice, in the inter-proximal and inter-radicular areas (Figures 1a,b), as well as in buccal and lingual alveolar crests relative to WT mice (Figures 1c,d). We performed eight measurements of the distance between the alveolar bone crest and cervical enamel of the mandible, in the areas facing each molar, for all mice (*n* = 5 in each group) (lines in Figures 1a,b). The mean values were significantly higher in R^Tg^ than WT mice, for all areas measured (Figure 1e). In addition, this increase was greater in transgenic females than males.

### Rank overexpression increases the number of trap-positive cells at the surface of alveolar bone, dentin, and the cementum

**Figure 2:**
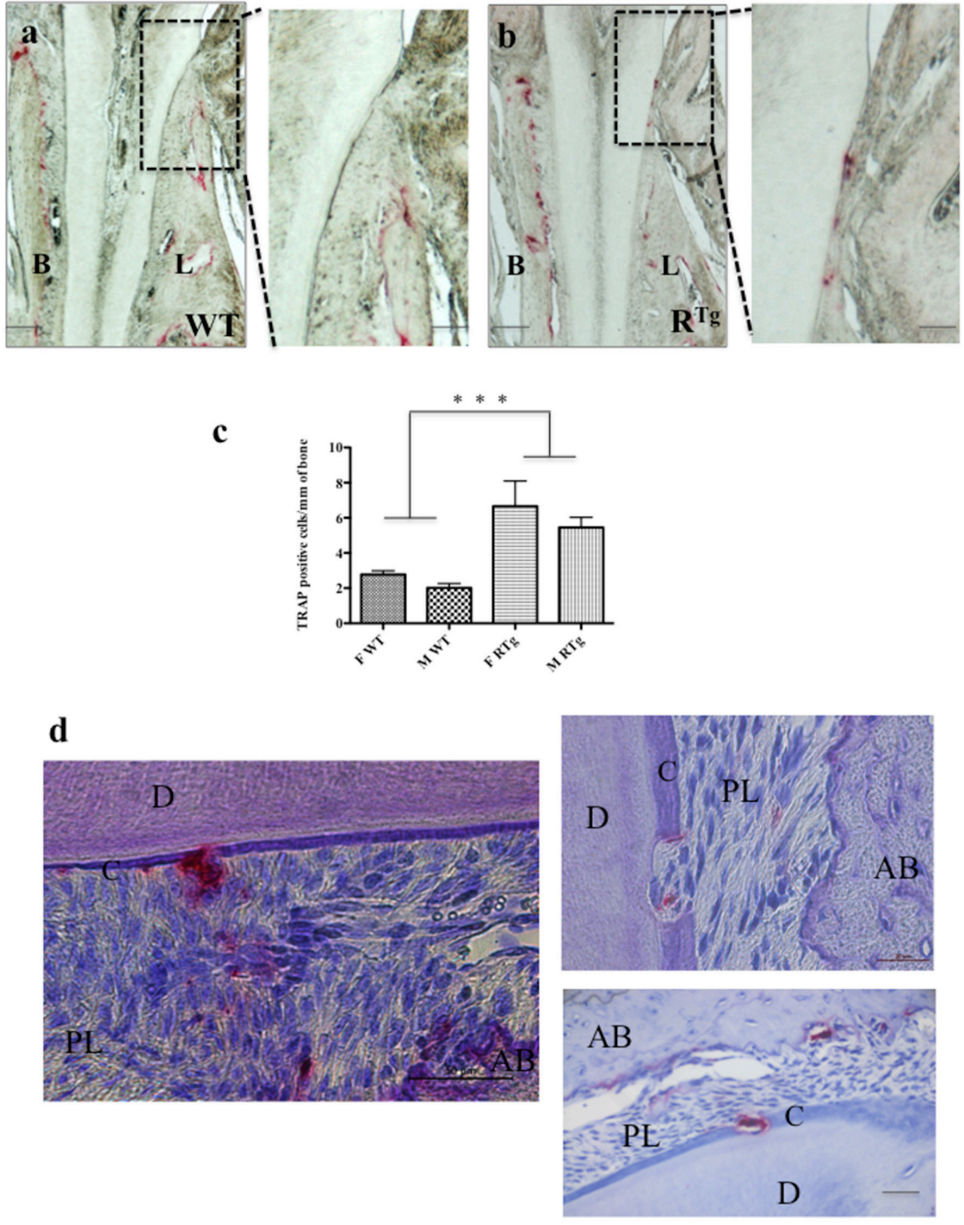
**TRAP positive cells in 5-month-old mice**. TRAP histo-enzymology performed on frontal sections **(a,b)** enabled visualization of a large increase in the number of TRAP positive cells (red staining) on both buccal (B) and lingual (L) surfaces of the alveolar bone of R^Tg^ mice (*n* = 10) relative to that of WT mice (*n* = 10). Magnifications of the lingual cervical dentin region (dashed rectangles in **a,b**) showed the presence of numerous TRAP positive cells on the dentin surface, almost exclusively in R^Tg^ mice. Quantification of TRAP-positive cells **(C)** on alveolar bone surfaces of WT and R^Tg^ females (in green) and males (blue) showed a significantly (^***^p < 0.001) higher number of cells respectively in both male (M) and female (F) R^Tg^ mice than in WT mice. Similar numbers of TRAP positive cells were visible at the cementum surface and in resorption lacunas through the cementum and dentin **(d)** in the root area of R^Tg^ mice. AB, alveolar bone; D, dentin; C, cementum; PL, periodontal ligament. Scale bars correspond to 10 μm in **a,b**, 20 μm in the enlarged **a,b** and right photograph in **d**, and 50 μm in the left photograph in **d**.

We performed TRAP histo-enzymology on the mandibles of adult 5-month-old WT and R^Tg^ mice (Figure 2). We observed a higher number of TRAP-positive cells at the alveolar bone surface of R^Tg^ than WT mice (Figures 2a,b). Quantification of the TRAP positive-cells relatively to the bone surface showed their number to be 2.4-fold higher in both male and female transgenic mice than in WT mice, and the difference was significant (Figure 2c). The larger standard deviation observed in the female R^Tg^ mice corresponded to the greater observed inter-individual variation, specifically in the root furcation area. The presence of TRAP-positive cells was also clearly visible at the surface of both the cervical dentin (enlargements in Figures 2a,b) and cementum (Figure 2d) in the R^Tg^ mice. Resorption lacunas in the cementum and part of the dentin were also evident, indicating an already advanced resorption process (Figure 2d).

### Rank overexpression leads to gradual modification of gingival epithelial attachment

**Figure 3:**
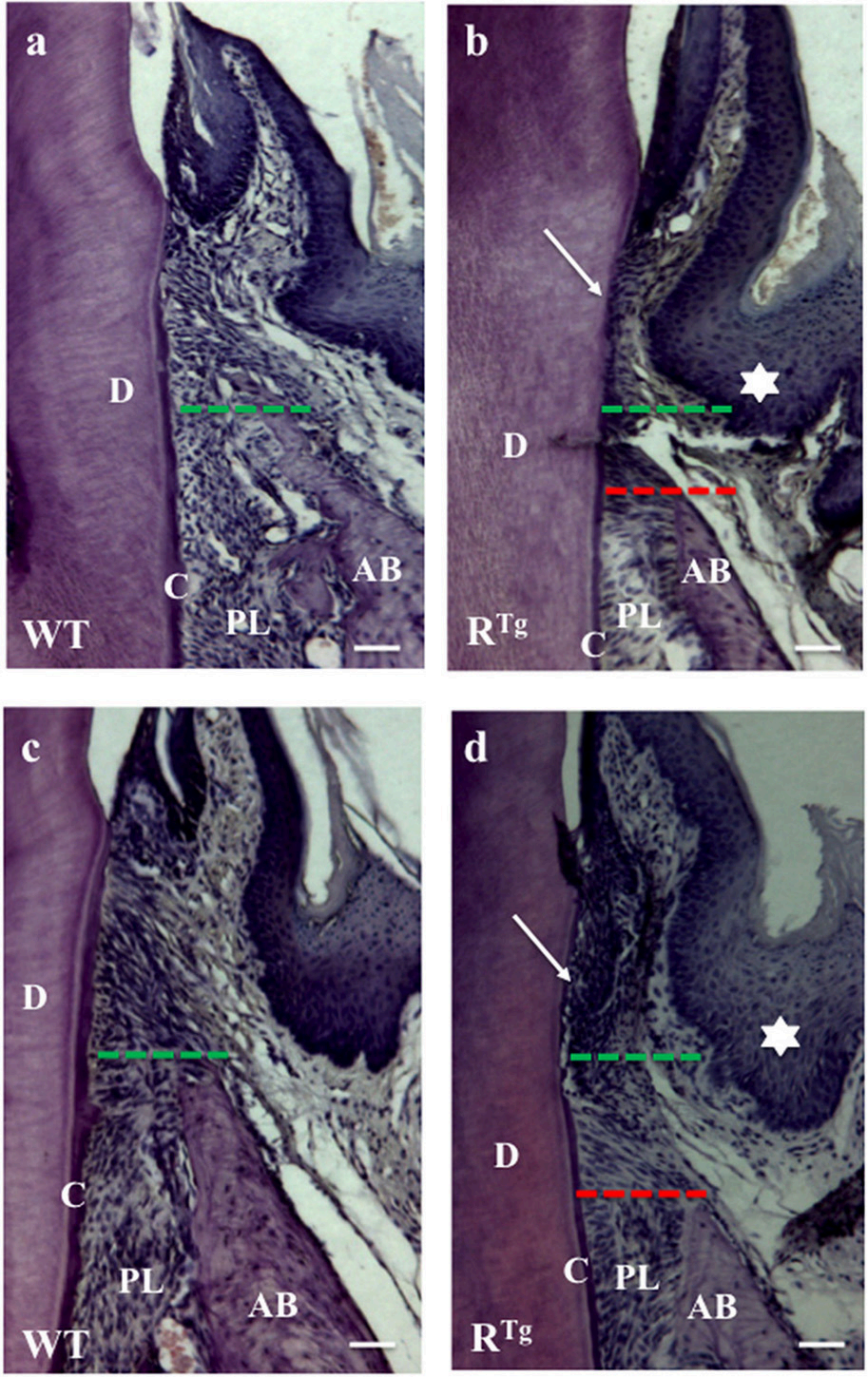
**Hematoxylin-Eosin staining of alveolar bone and gingival epithelium**. Frontal sections were cut in the molar plane 5 and 6-month-old WT **(a,c)** and RTg **(b,d)** mice, and a gradual reduction of the alveolar bone height was detected (green and red lines). This reduction was associated with the detachment and disruption of the epithelial junction (arrows in **b,d**). We also observed thickening of the gingival epithelium in R^Tg^ mice (stars in **b,d**). AB, alveolar bone; D, dentin; C, cementum; PL, periodontal ligament. Scale bars correspond to 20 μm.

Hematoxylin-eosin staining of adult 5-month-old WT and R^Tg^ mouse heads (Figures 3a,b) showed a gradual loss of junction attachment of the gingival epithelium (arrows) following its lengthening in association to alveolar bone loss in the R^Tg^ mice (green and red dashed lines in Figures 3a,b). Progression of the alterations was demonstrated by the more pronounced phenotype in older R^Tg^ mice at 6 months of age (Figures 3c,d).

### Early increase in epithelium thickness and hyperplasia in R^Tg^ mice

**Figure 4:**
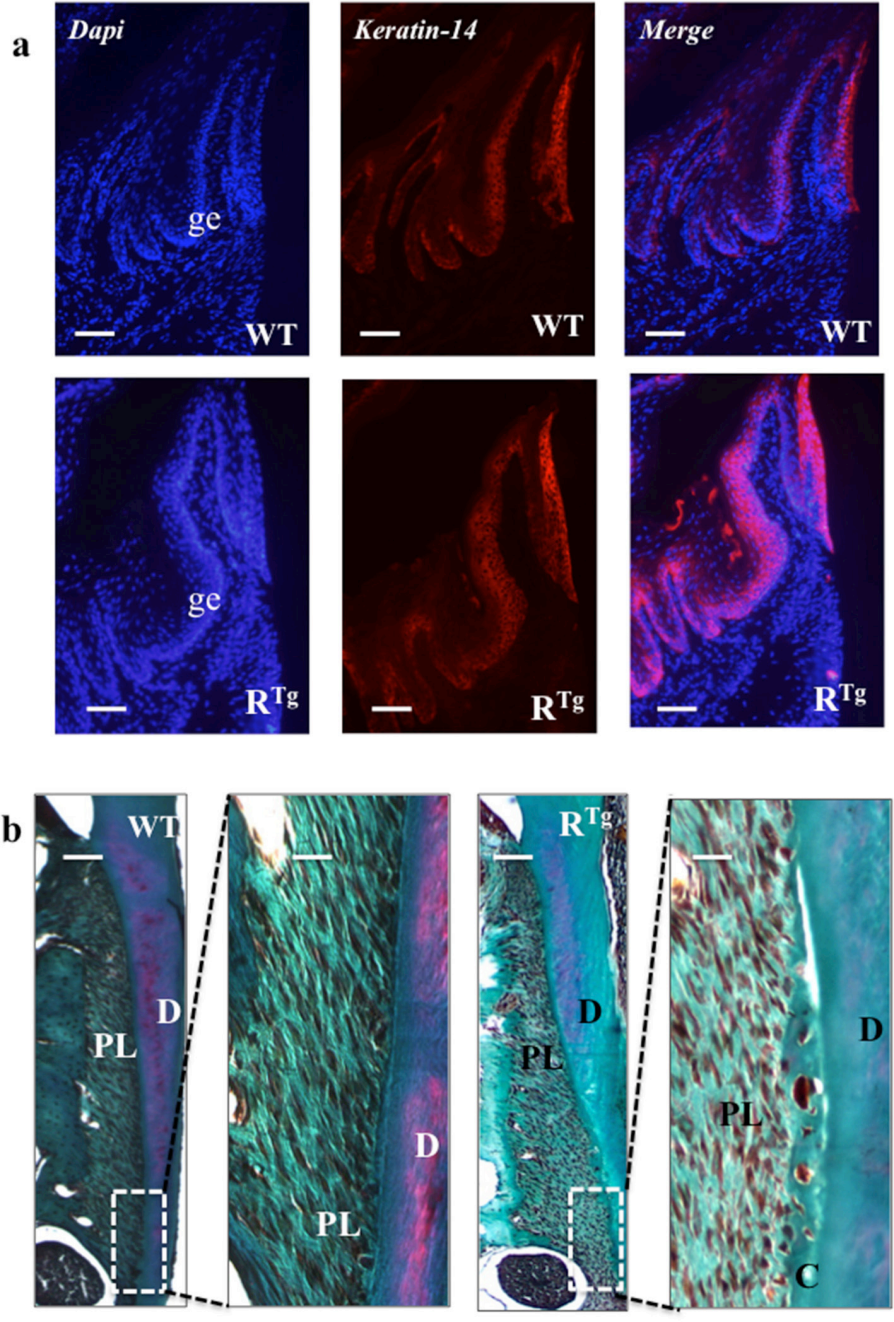
**Histological analysis of gingival epithelium and periodontal ligaments at 25 days**. At this age, an important thickening of gingival epithelium was already clearly visible in R^Tg^ mice relative to WT mice **(a)** whereas we observed no difference in the structure of the periodontal ligament **(b)**. Masson trichrome staining showed no difference regarding periodontal tissues or ligaments between WT and R^Tg^ mice. AB, alveolar bone; D, dentin; C, cementum; PL, periodontal ligament. Scale bars correspond to 50 μm in **a**, 10 μm in **b** and 50 μm in enlarged **b**.

**Figure 5:**
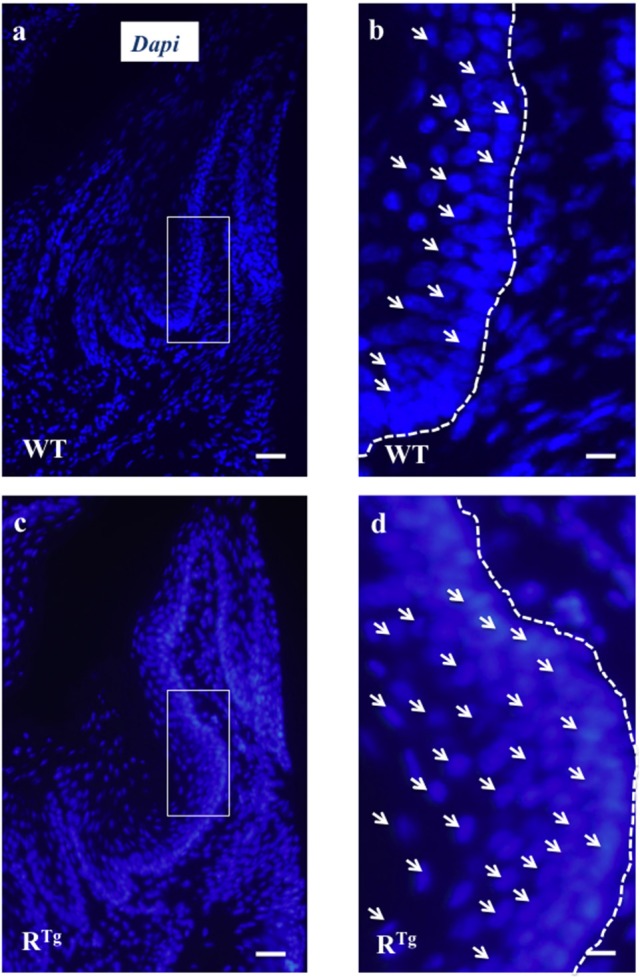
**Evidence of gingival epithelium hyper-cellularity in R^**Tg**^ mice based on numbering of DAPI stained cell nucleus**. Enlargement view of similar region of the gingival epithelium (rectangles in **a,c**) evidenced a two times augmentation of the number of cells (arrows) in the gingival epithelium of R^Tg^ mice comparatively to WT mice **(b,d)**. Scale bars in **A,C** correspond to 100 μm and in **b,d** to 25 μm.

**Figure 6:**
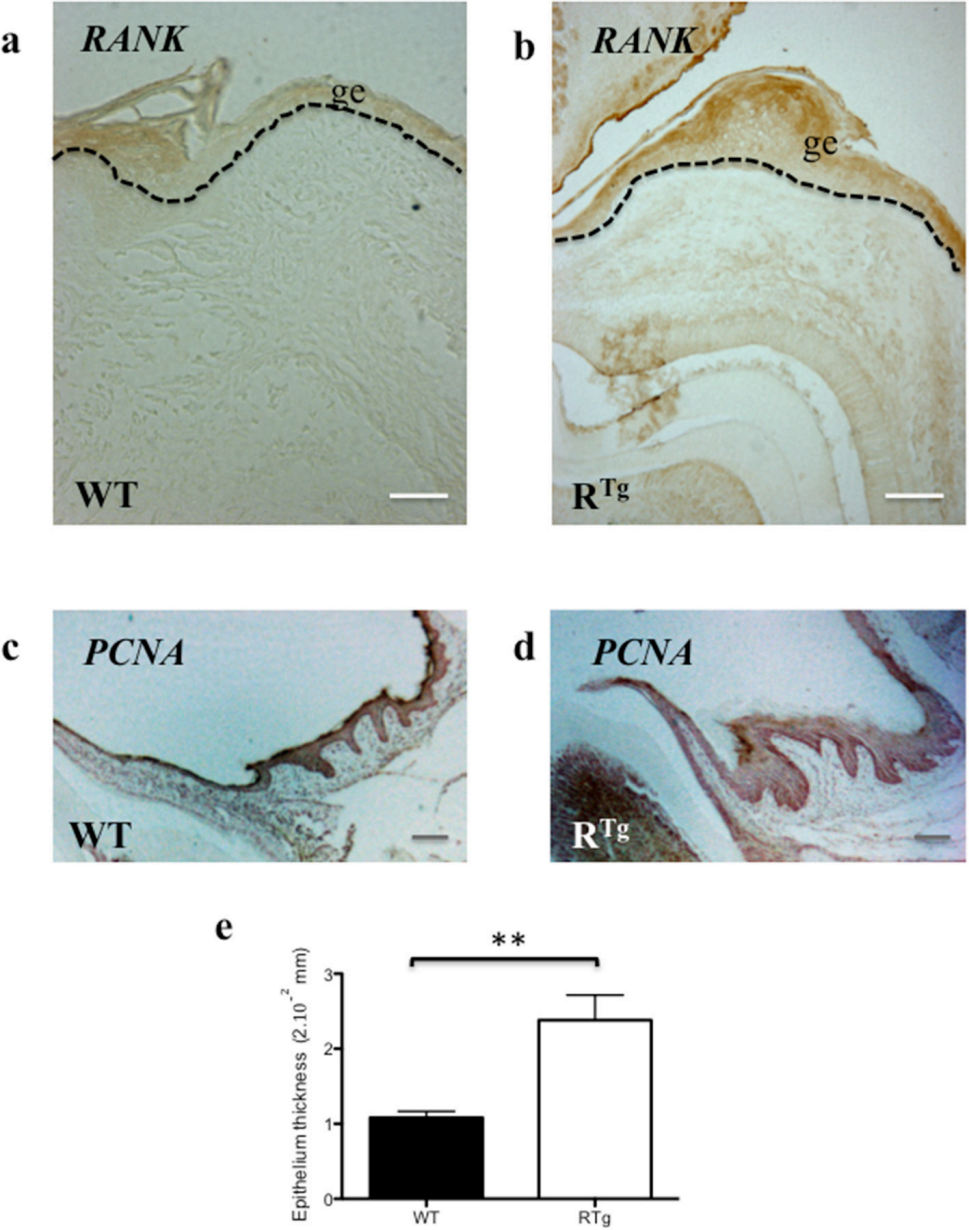
**Immuno-histochemistry analyses of RANK and PCNA expressions comparatively in WT and R^**Tg**^ mice**. The RANK expressing gingival epithelium thickness was already increased in 5 day-old transgenic mice comparatively to WT **(a,b)**. PNCA staining performed on sections of at 25 day-old WT and R^Tg^ evidenced that gingival epithelium thickness increase is associated to an increase of proliferative cells number **(c,d)**. Measurement of the epithelium thickness performed on these sections evidenced a significant increase (^**^p < 0.01) in the R^Tg^ mice **(e)**. ge, gingival epithelium. Scale bars in **a,b** correspond to 50 μm and in **C,D** to 20 μm.

The gingival epithelium was thicker in adult 5- and 6-month-old R^Tg^ mice than in WT mice of the same age (Figures 3b,d). At a younger age corresponding to the end of the growing period (25 days), immunofluorescent labeling of the epithelial marker, keratin 14, already showed a greater thickness of the gingival tissues in the R^Tg^ mice (Figure 4a) corresponding to an increase number of epithelial cells has evidenced by Dapi staining at higher magnification (Figure 5). In very young animals (5 days) this increase thickness was already visible associated to RANK over-expression (Figures 6a,b). This significant increased thickness was associated to a higher proliferation of epithelial cells has evidenced by the PCNA staining (Figures 6c–e).

Masson trichrome staining of 25 day-old mice did not show differences in periodontal tissues or ligaments between WT and R^Tg^ mice (Figure 4b).

**Figure 7:**
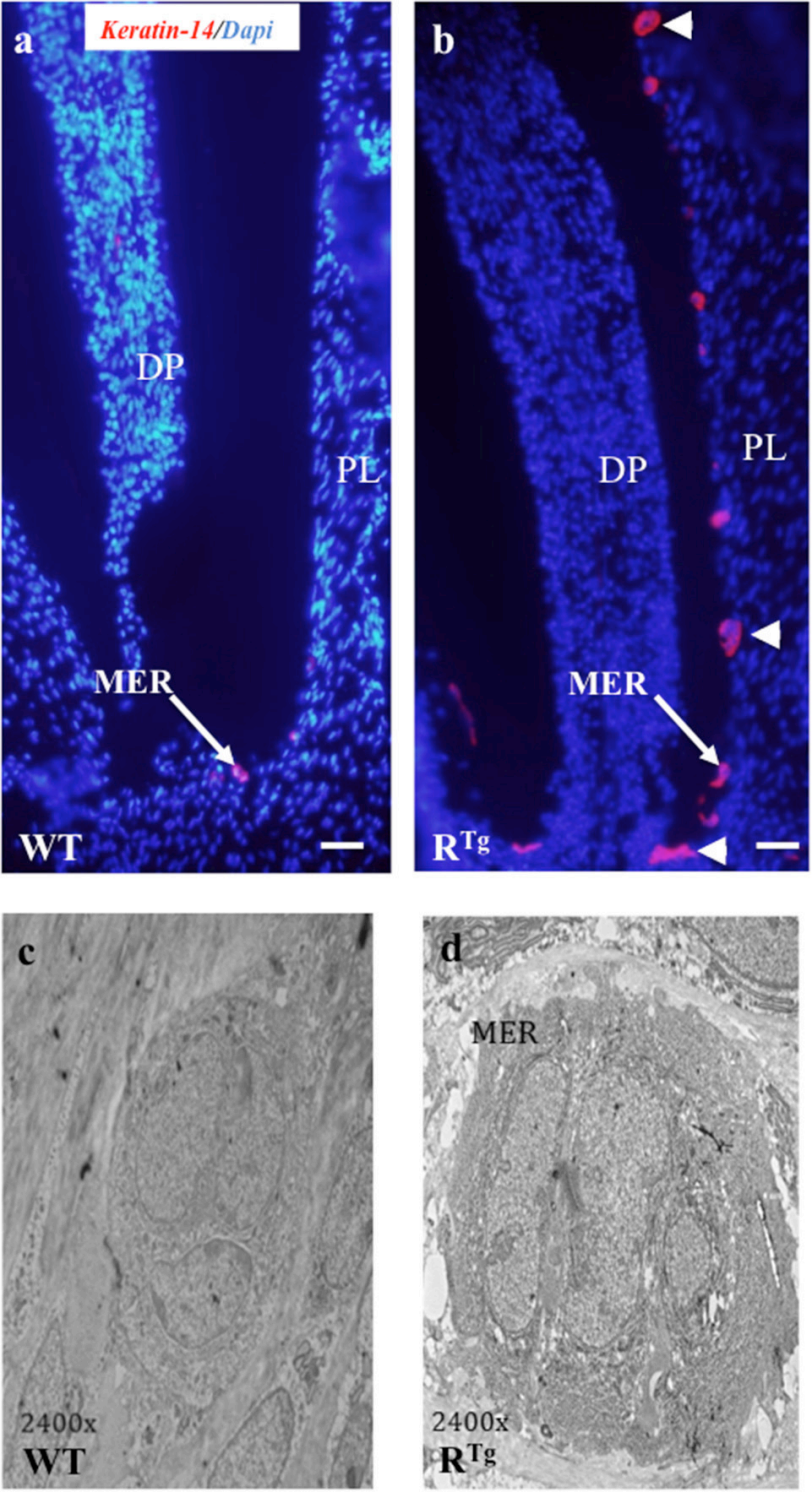
**Histological (Keratin 14 immunohistochemistry) and structural (TEM) analyses of Malassez epithelial rests (MER) in 25-day-old mice**. A higher number of MER was observed in R^Tg^ than WT mice (red staining in **a,b**) with most appearing to be hyperplasic and/or hypertrophic (arrow-heads in **b**). TEM analysis **(c,d)** showed that the hypertrophic appearance of MER was associated with an increased number of cells rather than a higher cell volume. DP, dental papilla; PL, periodontal ligament. Scale bars in **a,b** correspond to 50 μm. TEM magnification is 2400 X.

**Figure 8:**
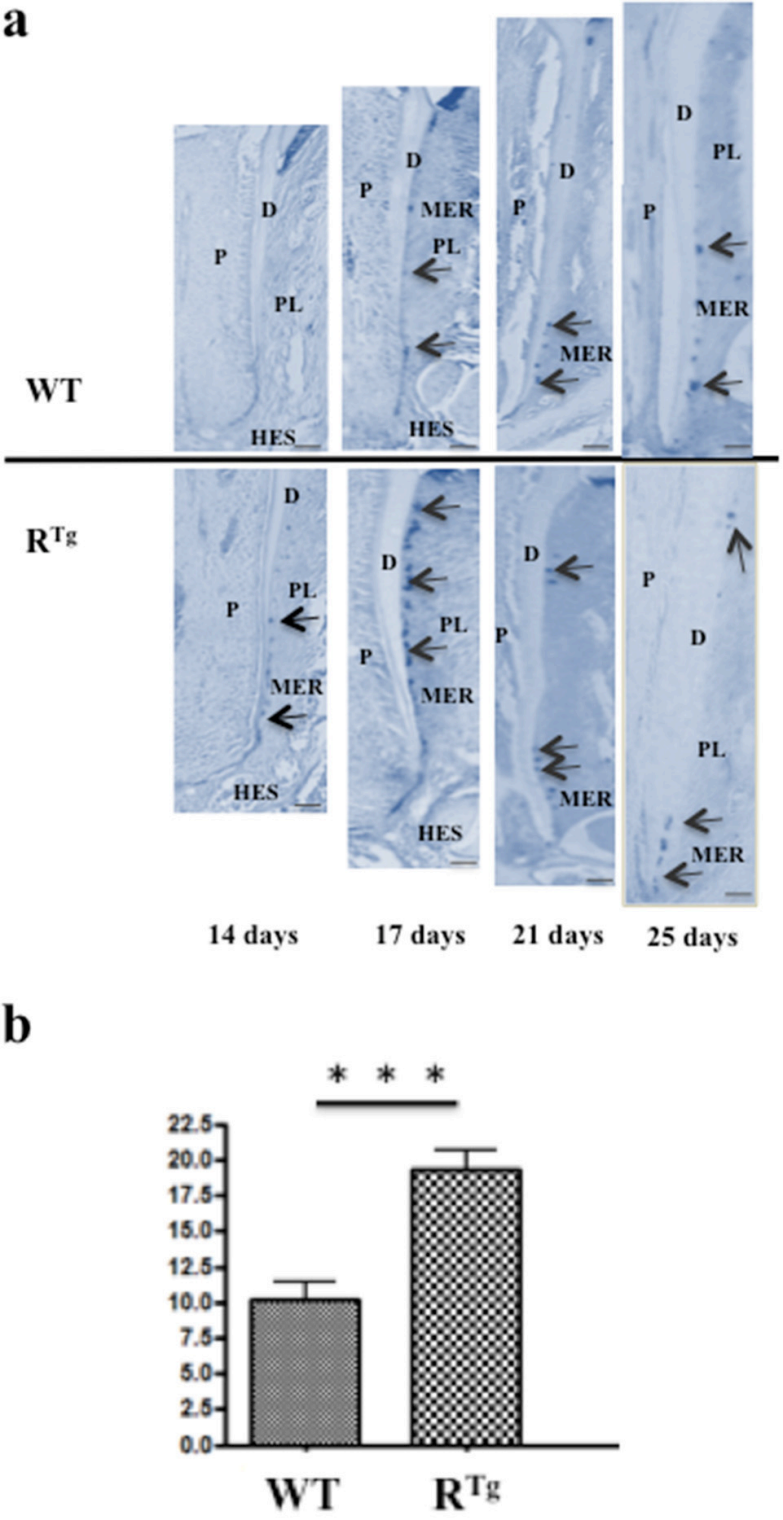
**Longitudinal analyses from days 14 to 25 of the number of Malassez epithelial rests (MER) based on Keratin 14 immunohistochemistry comparatively in R^**Tg**^ and WT mice**. Keratin 14 positive MER were numbered in equivalent areas of the mandible first molar roots of WT and R^Tg^ mice at 14, 17, 21, and 25 days-old (arrows in **a**). Obtained values were combined in a single statistic analysis in order to represent the median global variation of the MER number during the whole root elongation **(b)** showing a significant (^***^p < 0.001) increase in the R^Tg^ mice. P, pulp; D, dentin; C, cementum; PL, periodontal ligament; HES, Hertwig epithelial root sheath. Scale bars correspond to 10 μm.

In addition to the greater gingival epithelium thickness observed in young R^Tg^ mice, we also observed a higher number of Malassez epithelial rest (MER) cells (Figures 7a,b) with most showing hyperplasia (Figures 7b, 8a). Quantification of MER number (Figure 8b) evidence a significant higher number in R^Tg^ mice. TEM further supported the observed hyperplasia of MER in R^Tg^ mice that was associated with a higher number of epithelial cells rather than an increase in size (hypertrophy) of each epithelial cell (Figures 7c,d).

## Discussion

Pd is an infectious disease of multifactorial origin (Page and Kornman, 1997; Albandar, 2005) characterized by gingival inflammation and alveolar bone destruction. Chronic exposure to components of the oral bacterial flora, in particular Gram-negative anaerobes, remains the major etiological factor (Haffajee et al., 2008). However, bacteria do not cause the destruction of periodontal tissues alone, but also stimulate an inflammatory immune response that is involved in the destruction. Thus, a cascade of biochemical and cellular events take place in the pathological progression leading to Pd, with the ultimate disruption of connective and bone tissue homeostasis (Hajishengallis and Sahingur, 2014). The local inflammatory response may be either attenuated or amplified by several risk factors of genetic or environmental origins, as well as patient lifestyle (Albandar, 2005; Nikolopoulos et al., 2008).

### Pd risk factors

Genetic factors, considered influencing the host response and for which a relationship with Pd has been established, fall into two broad categories. The first include the obvious genetic factors responsible for genetic diseases in which periodontal manifestations are present, such as Papillon-Lefevre syndrome (Bimstein et al., 1990) and leukocyte adhesion deficiency (Dababneh et al., 2008). The second includes more discrete genetic factors that do not noticeably affect one's general wellbeing but which nevertheless predispose the individual to Pd (Kinane and Hart, 2003). In this category, the role of certain gene polymorphisms in determining individual susceptibility to Pd has been reported. The most studied genes encode inflammatory cytokines, such as IL6, IL1, IL10, and TNF alpha (Huynh-Ba et al., 2007; Nibali et al., 2007). Several studies have also shown associations between the expression of RANKL/RANK/OPG triad elements and Pd (Cochran, 2008; Giannopoulou et al., 2012). Indeed, gingival samples from periodontal lesions have been shown to have significantly higher RANKL and lower OPG mRNA levels than those of healthy subjects (Bostanci et al., 2007). Similarly, abundant RANKL-positive cells were found in the inflammatory epithelium and connective tissues of gingiva of patients with chronic periodontitis (Bhuvaneswarri et al., 2014). Our results showed that RANK overexpression in cells of the monocyte/macrophage lineage and in gingival epithelium cells in mice led to the loss of alveolar bone height and an increase in the number of TRAP-positive cells in alveolar bone, reflecting the over-activation of osteoclasts and accelerated resorption whose origin may be the inflammation of the gingival epithelium that precede any sign of bone lost (Figures 4, 6). Thus, this genetically achieved experimental model of a Pd-like phenotype supports an imbalance in RANKL/RANK/OPG signaling as a major contributing factor for Pd, whether it is systemic or localized to periodontal tissues, regardless of its origin (bacteria or genetically inherited).

Osteolytic bone diseases directly associated with an increased RANK/OPG ratio may result either from RANK over-activation/expression, such as in familial expansile osteolysis (FEO, OMIM #174810) and Paget disease of Bone 2—early onset (PDB2, OMIM #602080), or from decreased OPG expression/function, such as in Paget disease of Bone 5—Juvenile-Onset (PDB5, OMIM #239000). Such pathologies have been associated with early and progressive loss of alveolar bone height, severe root resorptions, premature exfoliation of the teeth, and disturbances of periodontal homeostasis and alveolar bone remodeling (Reddy, 2004; Nuti and Ferrari, 2015), all characteristic signs of Pd.

We previously found that R^Tg^ mice exhibit early tooth eruption and accelerated tooth root elongation after an increase in the number of osteoclasts surrounding the tooth during growth. Accordingly, we observed more osteoclasts in alveolar bone of adult R^Tg^ mice than in that of adult WT mice in the present study. Additionally, we showed an increase in the number of TRAP-positive cells and consequently, a greater loss of alveolar bone height in female than male R^Tg^ mice. Female estrogen levels decline with age and this gradual estrogen deficiency is associated with increased production of pro-inflammatory cytokines that activate bone resorption (Riggs, 2000; Zhao et al., 2012), and reduced OPG and increased RANKL production by osteoblasts, two direct target genes of estrogens (Bord et al., 2003). More studies must be conducted to show that the increase in the number of osteoclasts in alveolar bone induced by RANK over-expression is also dependent on sex steroid hormones.

Concerning superficial periodontal tissue, the gingival tissue in R^Tg^ mice was thicker than that of WT mice in early life. This increase was associated with the involvement of RANK in gingival epithelium homeostasis, as previously established for the epidermis (Duheron et al., 2011). Indeed, RANK is expressed by keratinocytes of the epidermal basal layer and is activated by RANKL. Moreover, the expression is much higher in supra-basal layer keratinocytes of R^Tg^ than WT mice. In this transgenic mouse line, the hyper-proliferation of keratinocytes was shown to be independent of immune system activation, but instead a cell-autonomous process (Duheron et al., 2011). The gingival and MER hyperplasia observed in the R^Tg^ mouse may also be autonomous or indicative of a local inflammatory process exclusively related to RANK overexpression in epithelial cells (Figure 6). The hypothesis is that RANK overexpression in epithelial cells stimulate the proliferation and block the final differentiation of the cells as already describe for epithelial cell of the mammary gland (for review: Sigl and Penninger, 2014) or from the skin (Duheron et al., 2011). An anti-apoptotic effect may also be present as observed in breath carcinomas (for review on RANK/RANKL and cancer Renema et al., 2016). Moreover, over-expression of the same inflammatory cytokines than those previously reported in R^Tg^ mouse skin (Hess et al., 2012) and alveolar bone (Castaneda et al., 2011) may be present in the gingival epithelium, for instance CXCL10, CXCL11, and CXCL13, and take part to the periodontal pocket formation which constitutes a pathognomonic sign of the Pd.

In summary, the overexpression of RANK and consecutive osteoclast over-activation and epithelium inflammation may be engaging factors for Pd.

### Pd and pathological resorption of mineralized dental root tissues share the same risk factors?

In contrast to bone tissue, the resorption of mineralized dental tissues is pathological, except for physiological root resorption accompanying deciduous tooth loss in humans. Tooth resorption may occur in different contexts, such as infection or trauma. Such resorption may be internal and/or external, superficial or deep depending on the nature of the stimulus and the site of irritation. Osteoclasts *per se* are cells that are responsible for mineralized dental tissue resorption. This cellular process is the same throughout the body, regardless of the resorbed tissue or the origin/type of cells involved. Thus, some authors include osteoclastic cells that resorb mineralized dental tissues under the generic name of odontoclasts, which share similar activities with bone osteoclasts (Ten Cate and Anderson, 1986).

Pathological dental root resorption is a frequent iatrogenic consequence of orthodontic treatments, resulting in the loss of cementum and dentin. Previous studies have shown the existence of genetic predisposition to root resorption, such as external apical root resorption (EARR; Wu et al., 2013). The genetic factors and their potential polymorphisms have been, and are still, actively investigated. As expected, the same factors are implicated in both Pd and pathological root resorption, as both processes are based on the recruitment and activation of osteoclasts (odontoclasts). For example, one polymorphism in the Il-1b gene has been associated with a 5.6-fold higher risk of developing EARR during orthodontic treatment (Aminoshariae et al., 2016). RANKL/RANK/OPG signaling is also implicated in pathological root resorption (Lossdörfer et al., 2002; Iglesias-Linares and Hartsfield, 2016). During severe external root resorption, periodontal ligament cells were shown to produce a large amount of RANKL, thus activating odontoclastogenesis and functional odontoclasts (Low et al., 2005). Cementoblasts are also a source of RANKL that may play a role in odontoclast activation (Yang et al., 2015). To date, no gene polymorphism of the RANKL/RANK/OPG triad genes has been reported as a risk factor for root resorption, but this hypothesis needs to be further explored. Our results with the R^Tg^ mice clearly associate such a signaling imbalance in the dental microenvironment with root resorption. RANK over-expression in this transgenic mouse line was already known to stimulate RANKL expression in the periodontal microenvironment (Castaneda et al., 2011) and promote the recruitment of osteoclasts (odontoclasts). Moreover, a decrease in OPG expression has been documented during aging (Cao et al., 2003), changing the RANK/OPG ratio and favoring the formation of resorptive cells (osteoclasts and odontoclasts), thus inducing the activation of bone and root resorption.

In conclusion, the Pd-like phenotype of the R^Tg^ mouse supports an imbalance of RANKL/RANK/OPG signaling as an important contributing factor to Pd. Our data also establish that such an imbalance is also a contributing factor for dental root resorption, another condition affecting the dento-alveolar bone complex.

## Ethics statement

Experiments were realized by people certified for animal experimentation following the protocols validated by the Ethic committee number 06 from Pays de La loire and autorized by the French Ministry of Agriculture: agreement #01083.02.

## Author contributions

BS, AB, FL, and BC have designed experiments. BS, DC, BC have performed experiments. BS, CM, SB, AB, FL, and BC have interpreted the results of the experiments. BS, CM, SB, AB, FL, and BC have written the manuscript and prepared the figures.

### Conflict of interest statement

The authors declare that the research was conducted in the absence of any commercial or financial relationships that could be construed as a potential conflict of interest.
